# Effect of Various Airborne Particle Abrasion Conditions on Bonding between Polyether-Ether-Ketone (PEEK) and Dental Resin Cement

**DOI:** 10.3390/polym15092114

**Published:** 2023-04-28

**Authors:** Pao-Chieh Lee, Tzu-Yu Peng, Tien-Li Ma, Kuan-Yu Chiang, Yuichi Mine, I-Ta Lee, Chang-Chiang Yu, Su-Feng Chen, Jian-Hong Yu

**Affiliations:** 1 School of Dentistry, College of Dentistry, China Medical University, Taichung 40402, Taiwan; 2 School of Dentistry, College of Oral Medicine, Taipei Medical University, Taipei 11031, Taiwan; typeng@tmu.edu.tw (T.-Y.P.);; 3 Research Center of Digital Oral Science and Technology, College of Oral Medicine, Taipei Medical University, Taipei 11031, Taiwanlongriver710918@gmail.com (C.-C.Y.); 4 Department of Materials Science and Engineering, National Taiwan University, Taipei 10617, Taiwan; 5 School of Dental Technology, College of Oral Medicine, Taipei Medical University, Taipei 11031, Taiwan; 6 Department of Medical Systems Engineering, Graduate School of Biomedical and Health Sciences, Hiroshima University, Hiroshima 734-8553, Japan; 7 Happy Dental Clinic, Taichung 42950, Taiwan

**Keywords:** polyetheretherketone, resin cement, airborne particle abrasion, abrasion parameters, shear bond strength, thermocycle aging test

## Abstract

The effects of alumina particle size and jet pressure on the bond strength of polyetheretherketone (PEEK) were examined to determine the airborne particle abrasion parameters with minimal effects on PEEK and to achieve optimal bond strength, as a reference for future clinical use. An alumina particle with four particle sizes and three jet pressures was used to air-abrade PEEK. Surface roughness (*R_a_*), morphology, chemical structure, and wettability were analyzed using a stylus profilometer, scanning electron microscope, X-ray diffractometer, and contact angle analyzer, respectively. The shear bond strength (SBS) of PEEK and dental resin cement was analyzed using a universal testing machine (*n* = 10). The failure modes and debonded fracture surfaces were observed using optical microscopy. Airborne particle abrasion increased the *R_a_* and hydrophobicity of PEEK and deposited alumina residues. The SBS generally decreased after thermal cycling. A large particle size damaged the PEEK surface. The effects of different particle sizes and jet pressures on the SBS were only significant in certain groups. Adhesive failure was the main mode for all groups. Within the limitations of this study, 110 μm grain-sized alumina particles combined with a jet pressure of 2 bar prevented damage to PEEK, providing sufficient SBS and bonding durability between PEEK and dental resin cement.

## 1. Introduction

In modern society, all age groups are paying increasing attention to aesthetic requirements. Therefore, aesthetic considerations must also be taken into account in dental treatment, and with this trend, metal-free treatments have become a current trend in dentistry today [1,2,3,4,5]. With innovations in material science and the popularization of digital technology, many new aesthetic materials have gradually been introduced into dentistry. These materials not only have considerable aesthetic properties, but also have better mechanical properties, such as strength, fatigue resistance, and water absorption resistance. Among these, the most popular new aesthetic material is zirconia, which has excellent mechanical properties and biocompatibility [6,7]. During the past decade, zirconia has been widely used in dental prosthetics, such as dental crowns, bridges, and implants [8,9].

The introduction of polyetheretherketone (PEEK) into dentistry in recent years has provided a new high-performance polymer that possesses natural and aesthetic properties [10,11]. PEEK consists of phenylene rings linked by ether bonds and carbonyl groups, and it offers buffering, shock absorption, fatigue resistance, abrasion resistance, and hydrolysis resistance [12]. With its stability in the oral cavity, resistance to degradation, and biocompatibility, PEEK has become a popular biomedical material [13]. Moreover, PEEK has density, elasticity coefficient, and compressive strength similar to those of human hard tissues, which make it a suitable option for dentures [14]. Digital production of PEEK-based dentures is also a convenient process with the ability to store data, allowing for easy reproduction of dental prostheses in the event of fractures [15,16,17]. In order to incorporate PEEK into clinical dentistry, it is necessary to compare its bonding properties with those of other dental materials [11,18]. Researchers have previously analyzed and evaluated the strength and durability of the bond between PEEK and polymethyl methacrylate (PMMA) for clinical applications [19,20]. The bond strength of PEEK (7.60–8.38 MPa) met the minimum strength requirement for clinical practice (>5.0 MPa) specified by the International Organization for Standardization (ISO) 10477 [21], but was still lower than that of the alloy material (11.73 MPa). The use of airborne particle-abraded PEEK with aluminum oxide (Al_2_O_3_), followed by the application of a thin layer of primer comprising functional methyl methacrylate (MMA) monomers, is now a generally recommended practice [22]. It is important to ensure that the primer layer is extremely thin to avoid affecting the bond strength. Optimal bond strength can be achieved through light curing using LED light with a wavelength of 370–400 nm for 90 s [23]. However, using aluminum sand with a particle size that is too large or using excessively high jet pressure for airborne particle abrasion can cause chipping or damage to dental prostheses. To avoid this, researchers recommend reducing the particle size or jet pressure as much as possible [24,25].

As introduced in the previous section, PEEK has gained popularity in dental applications due to its biocompatibility, mechanical properties, and radiolucency [10,11,12,13,14,15,16,17]. However, to fully understand its clinical implications and align it with evidence-based dentistry, further studies are needed to evaluate its suitability for dental practice, long-term behavior, and performance in complex oral environments. Additionally, research on the effects of different surface treatments on the bond strength between PEEK and dental cement is valuable for establishing clinical guidelines and optimizing its use in dentistry [11,18,19,20]. This study aims to investigate the impact of various airborne particle abrasion conditions on the bond strength between PEEK and dental resin cement. The study will firstly investigate the effects of airborne particle abrasion conditions, such as particle size and jet pressure, on the surface of PEEK and the bond strength between PEEK and dental resin cement. Secondly, the study will evaluate the airborne particle abrasion parameters that have minimal effects on the properties of PEEK to achieve optimal bond strength as a reference for future clinical applications. The study proposes two experimental hypotheses: the first is that different airborne particle abrasion conditions do not affect the surface characteristics of PEEK, and the second is that different airborne particle abrasion conditions do not affect the bonding between PEEK and dental resin cement.

## 2. Materials and Methods

### 2.1. Specimen Preparation

As a result of G power analysis, the number of samples was determined as *n* = 10 per group. A three-dimensional model of a disc-shaped specimen with a diameter of 10 mm and a thickness of 2.5 mm was designed by using computer-aided-designed software (SolidWorks corp., Dassault Systemes, Waltham, MA, USA) and was converted into a stereolithography file for transfer to a dental milling machine (DGSHAPE DWX-52DCI; Roland DG Corp., Shizuoka, Japan) to produce a total of one hundred twenty PEEK specimens. The materials used in this study are listed in Table 1. The specimens were polished with 600-grit silicon carbide abrasive paper (Govern Trading Co., Ltd., New Taipei City, Taiwan), cleaned with distilled water in an ultrasonic cleaner (KUDOS-SK5200 BTU; Lenon Instruments Co., Ltd., Taoyuan, Taiwan) for 15 min, and air-dried. This state was denoted the control group (CON).

### 2.2. Surface Treatment and Bonding Procedures 

A dental sandblaster (Basic Quattro IS, Renfert GmbH, Hilzingen, Germany) was used to abrade the surface of PEEK with alumina particles of four sizes (50, 90, 110, and 125 μm), three jet pressures (2, 3, and 4 bar) [26], and were vertically abraded 10 mm from their surface for 10 s, resulting in a total of 12 different airborne particle abrasion conditions. Subsequently, a clear acrylic ring with an inner diameter of 6 mm was applied to the PEEK surface to confirm the bonding area. The transparent resin cement was then injected into the acrylic ring and light cured for 1 min. Then the excess cement was removed and it was placed in an oven at 37 °C for 60 min to ensure that the cement was completely cured. Note that all the surface treatment and bonding procedures were conducted by the same trained person to ensure standardization.

### 2.3. Surfaces Analysis

The samples were observed using a field emission scanning electron microscope (FE-SEM; JSM-7800F Prime, JEOL Ltd., Tokyo, Japan) at an acceleration voltage of 3.0 kV and under a vacuum condition of 9.6 × 10^−5^ Pa. For each specimen, the average roughness (*R_a_*) of the surface within an area of 500 μm × 500 μm was obtained using a stylus profilometer (DektakXT, Bruker Taiwan Co., Ltd., Hsinchu, Taiwan). Three specimens (*n* = 3) were prepared for each experimental group, and three different areas were evaluated for each sample to determine three *R_a_* values; the average value was calculated to represent the final *R_a_* value of each sample. The wettability of each sample (*n* = 10) was determined using a contact angle analyzer (Phoenix Mini, Surface Electro Optics Co., Ltd. Gyeonggi-do, Republic of Korea). The surface free energy (SFE) was calculated on the basis of the Girifalco-Good-Fowkes-Young theory. Changes in the surface lattice of PEEK after airborne particle abrasion under different conditions were investigated using a high-resolution X-ray diffractometer (HR-XRD; D8 SSS, Bruker Taiwan Co., Ltd., Hsinchu, Taiwan) under grazing-incident diffraction conditions to record between 15° and 60° with a counting scan speed of 4° per minute.

### 2.4. Shear Bond Strength

Half of the specimens (*n* = 10) were placed in a distilled water bath at 37 °C for 24 h, and the other half (*n* = 10) were placed in a thermocycler with 5,000 cycles of cold water at 5 °C and hot water at 55 °C, according to the ISO 10477 specification [27]. After all steps were completed, a universal testing machine (JSV-H1000; Algol Instrument Co., Ltd., Taoyuan, Taiwan) was used to apply a shear force at the bonding interface of the specimen and at the acrylic ring at a crosshead speed of 1 mm/min until the interface fractured. The shear bond strength (SBS) of each specimen was calculated to analyze the strength of bonding between the specimen and dental resin cement. After the SBS test, the debonded fracture surface of each specimen was observed with a dental microscope, and the failure mode, including adhesive failure (A failure), cohesive failure (C failure), and a combination of cohesive and adhesive failure (AC failure) was defined according to the debonding condition. Finally, the representative morphology of the debonded fracture surface was observed using an optical microscope (BA210; Motic China Group Co., Ltd, Xiamen, China).

### 2.5. Statistical Analysis

The mean and standard deviation of all data were calculated using statistical software. First, the normal distribution and homogeneity of variance for all values were analyzed using the Shapiro-Wilk test and Levene’s test. Parametric analysis was performed because all the data in this study were normally distributed. The SBS of PEEK and dental resin cement after treatment with different airborne particle abrasion conditions and thermal cycling was analyzed by one-way analysis of variance and compared using Tukey’s honestly significant difference test. All statistical analyses were performed using standard statistical software (IBM SPSS version 19.0, Armonk, NY, USA), and the significance level was set at 5%.

## 3. Results

**Figure 1:**
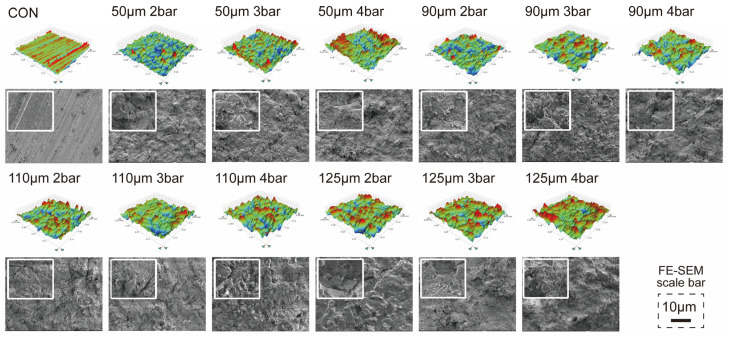
Surface topography (500 × 500 μm) and FE-SEM micrographs for each airborne particle abrasion condition. The scale bar is 10 μm (500× magnification); the small zoomed in diagram at the upper left corner of each FE-SEM micrograph shows a tenfold magnification (5000× magnification). CON is the control group that was ground with 600-grit silicon carbide abrasive papers only.

Figure 1 shows the micromorphologies and topographies of the untreated and air-abraded specimens, and the results are presented in Table 2. The topographic maps show that the control group (CON) had linear scratches, whereas all the airborne particle-abraded groups had similar disorderly patterns. The FE-SEM images of the samples that were airborne particle-abraded with 50, 90, and 110 μm particles showed similar surface morphologies. In particular, at higher jet pressure (4 bar), there were clear patterns of alumina particles stuck on the PEEK specimens. The groups that were airborne particle-abraded with 125 μm particles had greater morphological differences, where a bubble-shaped structure appeared for the airborne particle-abraded groups at a jet pressure of 2 bar, whereas the airborne particle-abraded groups with jet pressures of 3 and 4 bar had relatively flat surfaces. According to the quantitative analysis data shown in Table 2, except for the airborne particle-abraded samples with particle sizes and jet pressure combinations of 90 μm 2 bar, 90 μm 3 bar, 110 μm 3 bar, and 110 μm 4 bar, the *R_a_* of PEEK airborne particle-abraded samples under the other conditions increased with increasing jet pressure or particle size, but there was no significant difference among the groups (*p* > 0.05). Furthermore, the CON group had a significantly lower *R_a_* value (*p* < 0.05). 

The contact angle (CA) and SFE data for the specimens are listed in Table 2. All the specimens became significantly hydrophobic after airborne particle abrasion (*p* < 0.05). In the 50 μm group, the surface hydrophobicity increased with increasing jet pressure, whereas no such trend was observed in the 90, 110, and 125 μm groups. However, a comparison of the results at fixed jet pressure showed that the highest hydrophilicity was achieved in the 50 μm group (*p* < 0.05), whereas no significant differences were observed in the 90, 110, and 125 μm groups (*p* > 0.05). The SFE was significantly higher in the CON group than in all the airborne particle-abraded groups (*p* < 0.05) and significantly higher in the 50 μm group among the airborne particle-abraded groups; there was no significant difference among the 90, 110, and 125 μm groups (*p* > 0.05).

**Figure 2:**
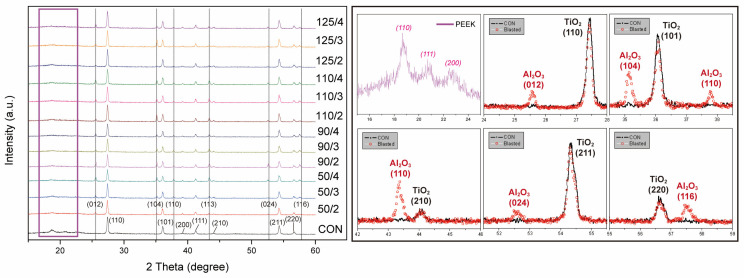
XRD profile of samples from each airborne particle abrasion condition. The legend in the left image shows the particle size (50, 90, 110, and 125 μm)/jet pressure (2, 3, and 4 bar). The right image is a zoomed in diagram of specific intervals.

Figure 2 shows the XRD profiles for all experimental groups, along with zoomed in diagrams of the local intervals. The most intense characteristic diffraction peaks of PEEK were centered at 2θ = 18.8°, 20.7°, 22.8°, and 28.8°, corresponding to the (110), (111), (200), and (211) Miller planes, respectively [28]. The XRD spectra of the CON group showed distinctive characteristic peaks at 2θ values of 27.5°, 36.2°, 39.3°, 41.3°, 44.1°, 54.4°, and 56.7°, corresponding to the (110), (101), (200), (111), (210), (211), and (220) planes of titanium dioxide particles, respectively [29,30]. The XRD patterns of all airborne particle-abraded groups showed additional characteristic peaks at 25.4°, 35.0°, 37.7°, 43.8°, 52.5°, and 57.4°, corresponding to the characteristic (012), (104), (110), (113), (024), and (116) peaks of alumina oxide, respectively [31,32].

**Figure 3:**
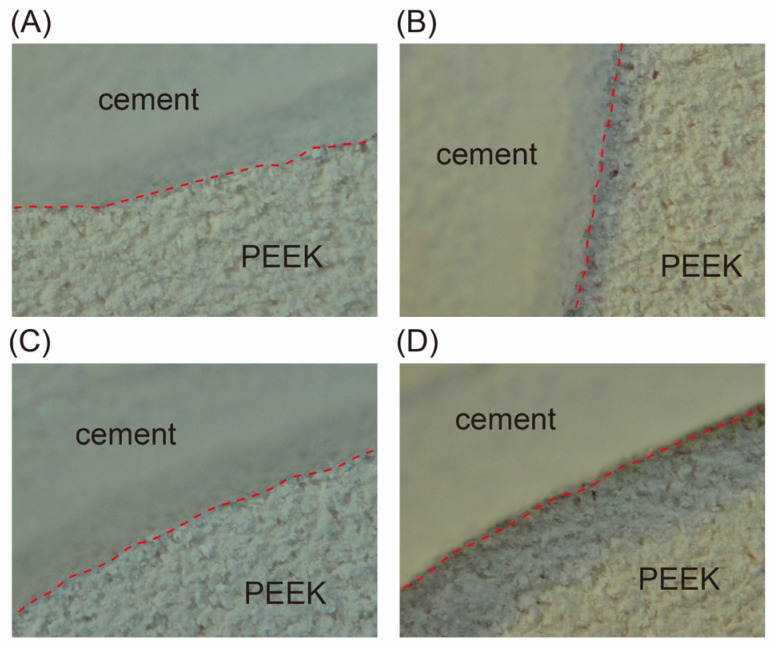
Optical micrographs (×40) of the representative fracture surfaces of AC failure. (**A**,**B**) are samples not subjected to thermal cycling, whereas (**C**,**D**) are samples subjected to 5,000 thermal cycles. The red dotted line is the interface between PEEK and dental resin cement.

Table 3 lists the SBS and failure modes of each group. Among the experimental groups that did not undergo thermal cycling, the SBS was significantly lower than that of the other groups (*p* < 0.05) and lower than the specification of ISO 10477 (>5 MPa) only for the 125 μm 2 bar group (4.73 MPa), whereas the SBSs of all other groups were greater than 6 MPa, with no significant difference (*p* > 0.05). After 5,000 thermal cycles, the SBS did not decrease for the 50 μm 2 bar group. The SBS of the 90 μm 2 bar, 110 μm 4 bar, and 125 μm 2 bar groups was below the ISO specification. In the intergroup comparison, the SBS was highest for the 110 μm 2 bar group (6.68 MPa), and was significantly higher than that of the 110 μm 4 bar group (4.37 MPa, *p* = 0.016) and the 125 μm 2 bar group (4.33 MPa, *p* = 0.013). A comparison before and after thermal cycling showed significant decreases in the SBS after thermal cycling (*p* < 0.05) for half of the experimental groups (50 μm 3 bar, 50 μm 4 bar, 90 μm 4 bar, 110 μm 3 bar, 110 μm 4 bar, and 125 μm 4 bar). Regarding the failure mode, A failure was most frequently observed (Table 3), and a clear fracture interface between PEEK and the resin cement was observed in the optical micrographs of the debonded fracture surface of the AC failure (Figure 3). 

## 4. Discussion

The PEEK material used in this study was combined with approximately 20 wt% titanium dioxide particles to adjust the color and mechanical strength [33]. As shown in the XRD profiles, the characteristic peak of titanium dioxide was apparent in the pattern of the CON group [29,30]. After airborne particle abrasion, the FE-SEM images showed lumpy alumina particles stuck on the PEEK specimens, and significant characteristic peaks of alumina oxide could be clearly seen on the planes with Miller indices of (104), (113), and (116) when the XRD profiles were partially zoomed in [31,32]. The spectrum of the pure PEEK powder shows characteristic peaks of the (110), (111), and (200) planes [28], whereas no significant peak was observed in the pattern of the PEEK material used in this study, which may be due to the addition of microfillers. The topographic diagrams obtained using the profilometer and the micromorphological diagrams obtained using FE-SEM showed that the airborne particle-abraded groups were structurally similar, except for the 125 μm 2 bar specimen, in which a bubble-like structure comprising a hybrid morphology was observed due to the exposure and destruction of titanium dioxide microfillers as a result of airborne particle abrasion. Quantitative analysis of the elemental distribution indicated that the weight percentages of carbon, oxygen, and aluminum atoms in the other groups were approximately 12, 50, and 38 wt%, respectively, while the elemental distribution of the group airborne particle abraded at 125 μm 2 bar had additional titanium dioxide, accounting for approximately 10 wt%. This also proves that overly large particles size can damage the PEEK surface. When the jet pressure was continuously increased to 3 or 4 bar, the surface gradually became rougher because of the continuous high-intensity damage, and the titanium dioxide microfillers gradually flattened. The bubble-like structure disappeared and the surface gradually flattened; thus, it was deduced that the jet pressure should not exceed 2 bar, as higher pressures will cause fundamental damage to the PEEK material. We also disproved the first experimental hypothesis because different airborne particle abrasion conditions affected the surface characteristics of PEEK.

Regarding the effects of particle size and jet pressure, the experimental results showed that the *R_a_* and hydrophobicity of the specimens increased, whereas the SFE of the specimens decreased with increasing jet pressure, except for the 50 μm group, and the remaining groups did not show significant differences. In addition, the effects of particle size on the surface of the PEEK were limited, with significant differences only between the 50 μm group and the other groups, while the differences among the air-abraded samples with other particle sizes were not significant. These results indicate that in airborne particle abrasion of PEEK, the use of particles smaller than 50 μm is unsuitable, which is contrary to the results of previous experiments carried out by other scholars using zirconia [26]. This is unlike zirconia, which is a high-strength crystalline ceramic, where the viscosity and abrasive resistance of the PEEK material [34,35] make it impossible for alumina particles that are too small to sufficiently roughen the surface. However, for the same reason, a certain jet pressure is required to attach alumina particles of sufficient size to the surface of PEEK.

Airborne particle abrasion with large particle size (125 μm) or with high jet pressure (4 bar) damages the PEEK surface without enhancing the SBS. Notably, the SBS of the 125 μm 2 bar group was significantly lower than that of the other groups (*p* < 0.05), regardless of whether PEEK was subjected to thermal cycling. This is because the microfillers mixed into the PEEK matrix are exposed, resulting in poor bonding; thus, the use of alumina particles larger than 110 μm is not recommended. The SBS decreased when the jet pressure increased to 4 bar for the 110 and 125 μm groups, and this decrease was more obvious after thermal cycling, which means that the use of both large particle size and high jet pressure will cause the SBS to decrease. However, in the small particle size groups (50 and 90 μm), the SBS increased with increasing jet pressure for the groups that were not subjected to thermal cycling. Nevertheless, these conditions were less effective for roughening the PEEK surface, and thus the SBS was still lower than that of the 110 μm 2 bar group, even when the jet pressure was increased to 4 bar. When observing the differences between before and after thermal cycling, for the airborne particle-abraded samples with the same particle size, all 4 bar groups showed a significant reduction of the SBS (*p* < 0.05), demonstrating that the material should not be airborne particle-abraded with an excessively high jet pressure. The current results are in line with other studies, where Stawarczyk et al. [36] found that the choice of adhesive system and air abrasion pressure had a significant impact on the bond strength between PEEK and veneering resin composite. Meanwhile, Kurahashi et al. [37] suggest that surface treatments are important for achieving a strong bond between PEEK and autopolymerizing resin. Herein, the second experimental hypothesis is also invalid because the airborne particle abrasion conditions affect the bonding between PEEK and dental resin cement. 

All the experimentally determined SBS values were greater than the ISO-specified value of 5 MPa [21]. However, the main type of failure identified through the failure mode and debonded fracture surface tests was adhesive failure. Tsuka et al. [38] reported that laser groove treatment significantly improved the shear bond strength between PEEK and a resin-based luting agent, with the highest result being 19.5 MPa. Kurahashi et al. [37] suggested that combining blasting and priming can improve the SBS of PEEK and autopolymerizing resin. Additionally, Peng et al. [20] found that surface treatment and primers have additive effects on the bond strength of PEEK material. In order to minimize experimental variables, primer or etching application was not included in the airborne particle abrasion procedure in this study, which may have affected the SBS results. Therefore, future experiments should evaluate the effect of priming or etching on the results. Additionally, PEEK can be enhanced by incorporating various microparticles, such as ceramic particles, to increase surface smoothness [39], or by adding glass or carbon fibers to improve the coefficient of elasticity [40]. Therefore, such modified PEEK must also be included in future experiments for evaluation.

## 5. Conclusions

Based on the findings of this in vitro study, the following conclusions were drawn:

1. Different airborne particle abrasion conditions affect the surface characteristics of PEEK. Large particle sizes (>125 μm) damage PEEK, while smaller ones (<110 μm) result in lower surface roughness, and jet pressure conditions do not make a difference.
2. Choosing conditions for airborne particle abrasion that minimize PEEK surface effects is recommended in clinical applications, as neither particle size nor jet pressure significantly impacted the SBS of PEEK and dental resin cement.
3. The optimal conditions for airborne particle abrasion of PEEK in clinical use are 110 μm alumina particles with a 2-bar jet pressure, which retain sufficient bond strength and durability while leaving PEEK undamaged.

## Figures and Tables

**Table 1:** Details of the materials used.

Materials	Main Composition *	Manufacturer	Lot Number
**Testing Materials**			
VESTAKEEP DC4450	80% PEEK with 20% filler including titanium dioxide and 1% pigment	Polyplastics-Evonik Corporation Ltd., Osaka, Japan	57781699
**Alumina Particle**			
Cobra	Al_2_O_3_, SiO_2_	Renfert GmbH, Hilzingen, Germany	2327409
**Dental Resin Cement**			
G-CEM LinkForce^®^	Paste A: bis-GMA, UDMA, dimethacrylate, etc. Paste B: bis-MEPP, UDMA, dimethacrylate, etc.	GC Corp., Tokyo, Japan	022009

* According to the manufacturer’s information. PEEK, poly(ether–ether–ketone); bis-GMA, bisphenol A-glycidyl methacrylate; UDMA, urethane dimethacrylate; bis-MEPP, bisphenol-A ethoxylate dimethacrylate.

**Table 2:** Surface roughness (*R_a_*), contact angle (CA), and surface free energy (SFE) for each group.

Conditions		*R_a_* (μm)	CA (degree)	SFE (mN/m)
CON		0.11 ± 0.03	92.79 ± 3.79	19.91 ± 3.96
50 μm	2 bars	1.39 ± 0.11	108.74 ± 7.55	10.73 ± 3.79
	3 bars	1.72 ± 0.03	110.00 ± 8.59	10.22 ± 4.03
	4 bars	1.80 ± 0.50	116.33 ± 11.42	7.80 ± 4.67
90 μm	2 bars	2.28 ± 0.07	136.66 ± 5.09	1.78 ± 0.85
	3 bars	2.17 ± 0.07	142.13 ± 1.85	1.01 ± 0.88
	4 bars	2.55 ± 0.05	136.95 ± 5.23	1.80 ± 0.88
110 μm	2 bars	2.41 ± 0.17	137.95 ± 1.45	1.50 ± 0.20
	3 bars	3.05 ± 0.01	136.85 ± 1.95	1.66 ± 0.29
	4 bars	2.94 ± 0.12	135.45 ± 3.69	1.91 ± 0.65
125 μm	2 bars	2.81 ± 0.04	138.81 ± 5.22	1.48 ± 0.67
	3 bars	3.64 ± 0.03	137.77 ± 2.84	1.55 ± 0.40
	4 bars	3.68 ± 0.33	132.91 ± 3.95	2.13 ± 0.53

All values are presented as average ± standard deviation. Conditions: PEEK samples airborne particle abraded with different jet pressures (2, 3, and 4 bar) and particle sizes (50, 90, 110, and 125 μm); CON, control group that was only ground flat using 600-grit silicon carbide abrasive papers.

**Table 3:** Mean values of bond strengths (MPa) and failure modes for each group.

Conditions		0 Thermocycles		5000 Thermocycles		S	Reduction
		Mean ± SD	Failure Mode A/AC/C	Mean ± SD	Failure Mode A/AC/C		
50 μm	2 bars	6.25 ± 1.13	10/0/0	6.31 ± 1.42	10/0/0		−0.96%
	3 bars	6.99 ± 1.15	9/1/0	5.26 ± 1.04	10/0/0	S	24.75%
	4 bars	7.36 ± 1.22	8/2/0	5.30 ± 1.70	10/0/0	S	27.99%
90 μm	2 bars	6.23 ± 1.23	10/0/0	4.97 ± 1.57	10/0/0		20.22%
	3 bars	6.82 ± 1.09	8/2/0	6.36 ± 1.31	10/0/0		6.74%
	4 bars	6.99 ± 0.75	10/0/0	5.25 ± 0.76	10/0/0	S	24.89%
110 μm	2 bars	7.43 ± 0.89	9/1/0	6.68 ± 0.76	10/0/0		10.09%
	3 bars	7.26 ± 0.56	7/3/0	5.64 ± 0.72	8/2/0	S	22.31%
	4 bars	6.04 ± 0.79	10/0/0	4.37 ± 0.37	10/0/0	S	27.65%
125 μm	2 bars	4.73 ± 0.66	10/0/0	4.33 ± 1.27	8/2/0		8.46%
	3 bars	6.58 ± 0.71	10/0/0	6.01 ± 1.34	10/0/0		8.66%
	4 bars	6.16 ± 0.87	9/1/0	5.02 ± 0.62	10/0/0	S	18.51%

Conditions, PEEK samples airborne particle-abraded with different jet pressures (2, 3, and 4 bar) and particle sizes (50, 90, 110, and 125 μm). SD, standard deviation. Failure mode: A, adhesive failure; AC, combination of cohesive and adhesive failure; C, cohesive failure. S, significant difference before and after thermocycling (*p* < 0.05). Reduction, rate of reduction.

## Data Availability

All data generated or used during the study appear in the submitted article.
